# Assessing nerve injuries in oral surgery: a survey-based study on prevention and management

**DOI:** 10.4317/medoral.26995

**Published:** 2025-03-23

**Authors:** Nelli Agbulut

**Affiliations:** 1 ORCID: 0000-0001-9860-5494. DMD, Specialist, Assistant Professor. Istanbul Medipol University, School of Dentistry, Department of Oral and Maxillofacial Surgery, Istanbul, Turkey

## Abstract

**Background:**

Understanding and mitigating iatrogenic nerve injuries, specifically IAN and LN, is crucial in the field of oral and maxillofacial surgery. These injuries may lead to profound sensory deficits, pain syndromes, and impaired quality of life for patients. This study aims to assess the prevention and management practices for inferior alveolar nerve and lingual nerve injuries among dental professionals. By gathering data through a survey, the study seeks to evaluate current experiences, awareness, and protocols, ultimately contributing to improved guidelines for nerve injury management.

**Material and Methods:**

This cross-sectional study utilized an online survey distributed via Turkish Dental Association to registered dentists and specialists. The predictor variables were the procedure types associated with nerve injuries. The main outcome variables were preventive measures and patient management strategies. Demographic profiling including age, years in profession, specialty, and type of current workplace were determined as covariates.

**Results:**

1477 respondents provided complete answers, with questions addressing demographics, nerve injury incidents, preventive measures, and management strategies. The most reported IAN and LN injuries were linked to dental implant surgery (*n*=1067), mandibular third molar surgery (*n*=958), and local anesthesia applications (*n*=459). Interestingly, more than 30% of participants reported no experience with nerve injuries. For preventive measures in tooth extraction, 281 respondents performed coronectomies, and in implant surgery over 80% of the participants evaluated tomographic images in high-risk cases. Most common management strategies for paresthesia included vitamin B12 (*n*=1093) and NSAIDs (*n*=1051). The use of gabapentin, and non-medical treatments like biofeedback and alternative therapies were rarely employed.

**Conclusions:**

The survey revealed a diverse range of practices regarding nerve injury prevention and management, emphasizing the need for evidence-based approaches and consensus guidelines. Understanding current practices for managing IAN and LN injuries can inform future guidelines, reduce complications, and improve patient outcomes in oral and maxillofacial surgery.

** Key words:**Nerve injury, oral surgery, survey, inferior alveolar nerve, lingual nerve.

## Introduction

Terminal branches of the trigeminal nerve are prone to injury during oral and maxillofacial surgical procedures. While most of these injuries do not necessitate further intervention due to spontaneous neurosensory recovery, some cases may result in significant functional deficits. These deficits can severely impair patients’ quality of life (1).

The mandibular division of the trigeminal nerve is more frequently injured compared to the ophthalmic and maxillary divisions during oral and maxillofacial surgical procedures. Among mandibular nerve’s branches, the most affected nerves are the inferior alveolar nerve (IAN) and lingual nerve (LN) (2,3). Neurosensory disturbances of these nerves often occur during third molar extractions, dental implant surgeries, orthognathic surgeries or endodontic treatments. Nerve damage may also occur less commonly during local anesthetic injections, ablative tumor surgeries, osteomyelitis or maxillofacial trauma. The risk of mandibular nerve injury across these procedures varies widely, ranging from 0.54% to 39% (1-3).

The literature lacks evidence and a consensus on the management strategies for inferior alveolar and lingual nerve injuries, whether surgical, medical, or psychological (4). Prompt diagnosis and management are essential to prevent long-term unwanted consequences and to avoid chronicity. Although each case should be individually assessed, meta-analyses suggest that early surgical repair may have better outcomes, though the optimal timing remains uncertain. Many experts advocate for timely intervention within a 90-day period (5).

Understanding and mitigating iatrogenic nerve injuries, specifically IAN and LN, is crucial in the field of oral and maxillofacial surgery. These injuries may lead to profound sensory deficits, pain syndromes, and impaired quality of life for patients (6). Despite advancements in surgical techniques and imaging technologies, the incidence of these complications persists, underscoring the need for comprehensive studies to identify risk factors, improve surgical outcomes, and enhance patient safety (7,8). Therefore, the aim of this study is to gather insights directly from dental professionals through a survey, aiming to elucidate current practices, experiences, and awareness regarding IAN and lingual nerve injuries. Consequently, the aim is to contribute valuable data that may inform guidelines, protocols, and training programs, ultimately reducing the occurrence and severity of these debilitating complications in clinical practice.

## Material and Methods

Ethical approval was obtained from the Non-invasive Clinical Research Ethics Committee of Istanbul Medipol University. (Decision No: 313 Date: 30.03.2023). This study follows the guidelines from the Helsinki Declaration.

- Study design/sample

This study was designed as a cross-sectional study where an online survey was conducted and distributed by sharing the survey link by email via the Turkish Dental Association to all registered dentists and specialists, which corresponds to more than 45,000 dentists and specialist according to the latest formal statistics from the Ministry of Health (9). Inclusion criteria encompassed being an actively practicing dentist or dental specialist. Exclusion criteria encompassed surveys with incomplete answers.

- Variables

Participants were asked nine questions. The predictor variables were the procedure types associated with nerve injuries. The main outcome variables were preventive measures and patient management strategies for either paresthesia after tooth extraction or implant surgery. Other variables were related to the demographic profiling including age, years in profession, specialty (if any), and type of current workplace. Participants were also questioned regarding their comments on coronectomy. Survey questions except those regarding demographic profiling are presented on Table 1.

- Data collection methods

Participants competed and submitted an online survey (Google Forms, California, USA) which consisted of multiple-choice questions, with certain questions allowing participants to select more than one response. The survey was available for three months, and reminder emails were sent one month after the initial invitation to boost response rates. Data from the completed surveys were transferred into Google Sheets for analysis.

- Data analyses

Statistical analyses were conducted using IBM SPSS v20 software. A minimum sample size of 104 participants was calculated based on 80% power and a margin of error of 5%. Results are presented as percentages, n (%).

## Results

The total number of respondents were 2203, 1477 of which provided complete answers. Demographic data of the participants are presented on Table 2.

Results regarding nerve injuries according to oral and maxillofacial surgical procedures are as follows: The three highest reported IAN or LN injuries were associated with dental implant surgery (*n*=1067), followed by mandibular third molar surgery (*n*=958) and applications of local anesthesia (*n*=459). Less commonly reported procedures included pathology related surgical interventions (*n*=192), endodontic treatment (*n*=47) or apical resection (*n*=36). More than 30% of participants reported that they never encountered IAN or LN injury.

When asked about coronectomy as a preventive procedure with regards to IAN injury, only 281 respondents mentioned performing coronectomy for indicated patients.

Participants’ choice of protocols for paresthesia management after tooth extraction revealed that 1093 of the participants prescribe vitamin B12 supplements, 1051 participants prescribe NSAID, whereas only 26 participants prescribe gabapentin. An even detailed analysis of the prescriptions revealed that participants who prescribe either B12 supplements or a NSAID, alone, are 784 and 791, in number, respectively. 242 in total, commented that they prescribe both a NSAID and a B12 supplement, whereas only 49 of the participants reported that they prescribe a combination of a B12 supplement and gabapentin. Only 18 of the participants reported that they used a combination of all three medications after patient-reported paresthesia.

Non-medical management strategies included transferring patients to either algology or neurology departments for further evaluation and treatment (*n*= 136) and biofeedback exercises (*n*=33). Other alternative options included laser, ozone therapy, radiofrequency or acupuncture, combined with either NSAIDs, B12 supplements or gabapentin by 63 participants, in total.

Forty-three participants mentioned that they employ a “watch-and-observe” strategy who choose to wait for a certain period until further intervention or treatment.

Choices of action in implant surgery for high-risk patients regarding nerve damage are summarized on Table 3.

When asked on their management strategies after patient-reported paresthesia following dental implant surgery, 736 participants commented not to undergo any surgical intervention but to start a medical treatment with either vitamin B12 supplements (*n*= 324) or corticosteroids (*n*= 309), or both (*n*= 103). Overall, only nine of the participants chose to observe the patients instead of any surgical or medical intervention. Moreover 262 participants wished to examine postoperative tomographic images before commencing any treatment. Fifty-two reported to ex-plant any dental implant(s) thought responsible for paresthesia. Fifty-one participants reported to ex-plant the suspected implant and to replace it with a shorter implant in length.

## Discussion

Neurosensory disturbances have an overwhelming effect on patients’ quality of life as well as bothersome legal issues between the dentist and the patient. Potentially devastating consequences of inferior alveolar and lingual nerve injuries for affected patients may attribute to inaccurate or insufficient radiological evaluation of the IAN and mental nerve, while also suggesting noncompliance in practice guidelines, and being deficient in risk assessment and in treatment planning in oral and maxillofacial surgery (10). Given these findings, along with the growing evidence, the use of 3D imaging in oral and maxillofacial surgery dentistry when operating near critical mandibular structures becomes crucial. This approach would enhance the accurate localization of these structures, enabling the selection of the optimal operational planning (8). Even though this study has revealed that more that 80% of the dentists and specialists in Turkey prefer to evaluate the tomographic images, a recent meta-analysis by commented that CBCT is not superior to panoramic imaging in avoiding neurosensory disturbances (10). Moreover, another study has pointed out that magnetic resonance imaging (MRI) is also being considered as an alternative to CBCT when assessing the three-dimensional relationship between the IAN and other structures also proving an advanced imaging modality that is radiation-free (11).

The findings from this survey have underscored the importance of inferior alveolar nerve (IAN) and lingual nerve (LN) injuries in oral and maxillofacial surgery, particularly in relation to dental implant procedures, mandibular third molar extractions, and local anesthesia applications. Data from this study have indicated that dental implant surgery is associated with the highest incidence of nerve injuries, followed by mandibular third molar surgery and local anesthesia applications. These results align with the literature highlighting the increased risk of nerve injuries with this procedures (1,6). Recent studies have corroborated that dental implant procedures are prone to complications involving IAN injuries, largely due to the proximity of the implant site to the inferior alveolar nerve canal as well as thermal injury due to drilling (1,12).

Interestingly, over 30% of respondents have reported never encountering IAN or LN injuries, which suggests variability in clinical experiences and possibly differing levels of procedural complexity or patient risk factors across practices. This variability may be attributed to differences in surgical techniques, patient anatomical considerations, and the implementation of preventive measures.

Participants have reported nerve damage due to endodontic treatments or apical resections in much lower incidences, a finding consistent with the lower complexity and reduced risk associated with these interventions. The results have been consistent with the incidences reported in the literature regarding endodontic treatments, but with a higher incidence of neurosensory deficit after periapical surgery (13-15). This may be attributed to the potential lack or unwillingness of reporting on errors and complications.

 A large number of participants have mentioned to perform coronectomies in appropriate cases. Although some authors are against coronectomies or advocate their use for a very limited number of indications, there is strong evidence that coronectomies are safer choices and good alternatives than total extractions when the apices of the teeth in close proximity to the IAN (16,17).

This survey has also highlighted a noTable variation in management strategies for post-operative paresthesia for tooth extraction. The majority of participants have reported prescribing vitamin B12 supplements, which have been supported by recent studies suggesting the potential benefits of B12 in nerve regeneration and recovery, especially by stimulating neuronal survival and remyelination (18,19). However, the prescription of gabapentin have been considerably less common, despite its established efficacy in managing neuropathic pain (6). This discrepancy may reflect locational differences in treatment preferences or a lack of awareness of gabapentin’s benefits in managing nerve-related symptoms. The observed preference for combining vitamin B12 with NSAIDs, rather than incorporating gabapentin, may reflect a more comprehensive approach to managing post-operative paresthesia, considering the prostaglandin synthesis inhibitory mechanism of NSAIDs that is beneficial for nerve recovery (20). It is noteworthy that only a small fraction of participants have used a combination of B12, NSAIDs, and gabapentin, suggesting that multi-modal approaches are not regionally adopted, whereas such an approach is accepted and reported in the literature (21). Non-medical management strategies, such as referring patients to algology or neurology departments, have been employed by a modest number of respondents. This approach aligns with contemporary advices for managing complex neurosensorial deficits that require specialized intervention (22,23). Conversely, the use of alternative therapies like low-level laser therapy, biofeedback, or acupuncture have been relatively rare, reflecting their less established role in standard practice despite some promising preliminary results (24). The “watch-and-observe” strategy have been employed by only three participants which may be attributed to the ongoing debate regarding the optimal management approach for post-surgical nerve injuries. While observational management may be appropriate in certain cases, especially when symptoms are mild or resolving spontaneously, it is critical to balance this with proactive treatment options to prevent long-term complications (25).

Regarding dental implant surgeries, data from this study have revealed a preference for medical treatment over surgical intervention, with vitamin B12 and corticosteroids being the most prescribed options. This approach is in line with recent studies suggesting that conservative management strategies, including medication and careful monitoring, may be effective in many cases (6,25). The current study has highlighted that a noTable number of participants prefers to review postoperative tomographic images before initiating any treatment, reflecting to a broader trend towards evidence-based decision-making in managing nerve injuries. Literature supports the decision to ex-plant implants, or to remove and replace them with shorter implants according to individualized treatment plans based on clinical judgment and imaging results. This approach helps accurately diagnose the cause of neurosensorial injury and plan appropriate interventions (26).

Overall, this study highlights the practice patterns of the participating dentists and specialists, as summarized on Table 4. Notably, despite the recognized preventive benefits of coronectomy in third molar surgery, its application remains relatively infrequent (27). This finding aligns with global trends, as documented in the literature, where there is a general hesitation toward performing coronectomy, with only a small proportion of clinicians feeling fully confident or adequately trained to determine when and how to carry out the procedure (28). In contrast, with regards to implant surgery, preventive measures such as the use of short implants and preoperative evaluation of tomographic images are more commonly practiced. The introduction of CBCT imaging in implantology has significantly enhanced preoperative planning, allowing for precise measurements, and the diagnostic data derived from these images have become increasingly fundamental to clinical decision-making globally, consistent with the findings of this study (29). Furthermore, current literature supports the use of short implants as a viable alternative to more complex surgical interventions, with an increasing body of evidence favoring their application in clinical practice (30). However, the underutilization of navigation systems indicates a potential area for enhancement. Regarding management strategies, the prescription of vitamin B12 and NSAIDs following tooth extraction, and vitamin B12 and corticosteroids after dental implant surgery, were identified as the most prevalent approached, mirroring the recommendations in the current literature (12,25). However, the preference for conservative and pharmaceutical approaches over surgical interventions to manage a nerve injury may suggest either a lack of confidence in surgical techniques or limited access to advanced training. Future advice may include promoting education on coronectomy as a preventive measure in third molar surgeries, highlighting the benefits of advanced surgical tools, like navigation systems in implant surgery, and encouraging the use of surgical techniques under correct indications to manage nerve injuries.

This study is not without limitations. The reliance on self-reported data may introduce response bias, as participants might overstate or underreport their experiences and practices. Additionally, the survey's cross-sectional design limits the ability to infer causality or long-term outcomes associated with different management strategies. The relatively small number of respondents who reports using alternative or novel treatments also suggests a need for further research to evaluate the efficacy and acceptance of these approaches on a broader scale. Future studies should aim to incorporate larger sample sizes, longitudinal follow-ups, and objective clinical outcomes to provide a more comprehensive understanding of the best practices for managing nerve injuries in oral and maxillofacial surgery. A final limitation of this study is the exclusion of incomplete responses, as missing data on key questions could have compromised the accuracy and reliability of the analysis. Despite these limitations, the findings of this study contribute to the ongoing dialogue on improving patient care and highlight areas for future research and refinement in clinical practice.

This survey reveals a diverse range of practices and preferences in managing IAN and LN injuries in Turkey, reflecting both the complexity of the issue and the need for continued research and guideline development. The findings underscore the importance of adopting evidence-based approaches and staying current with the advancements in treatment strategies to improve patient outcomes in oral and maxillofacial surgery.

## Figures and Tables

**Table 1:** Survey Questions except Demographic Profiling.

Survey Questions	Outcome variables
Procedures where you encountered Inferior Alveolar or Lingual Nerve Injuries (Check all that apply)	Mandibular third molar surgery
	Local anesthesia application
	Dental implant application
	Endodontic treatment
	Apical resection surgery
	Surgical intervention related to a pathology
	I have not encountered nerve injuries.
Do you perform coronectomy on tooth you consider to be at high risk of nerve injury during mandibular third molar surgery?	Yes
	No
What treatments do you prefer if a patient reports paresthesia after tooth extraction? (Check all that apply)	I monitor and wait.
	I prescribe NSAIDs.
	I prescribe vitamin B_12._
	I prescribe gabapentin.
	I recommend bio-feedback exercises.
	I plan a surgical intervention for patients reporting pain and paresthesia.
	I refer to neurology or algology departments.
	I opt for alternative options like laser, ozone therapy, radiofrequency, or acupuncture.
	I have not had a patient report paresthesia after tooth extraction.
Which of the following do you prefer in implant surgery with a high risk of nerve injury? (Check all that apply)	I use short implants.
	I evaluate tomographic examinations preoperatively.
	I apply corticosteroids before or after the procedure.
	I prefer navigation systems or guided surgical techniques.
	I use implant drills that utilize stoppers.
	I perform nerve repositioning or transposition.
What is your approach to patients reporting paresthesia after dental implant application?	I decide after tomographic evaluation.
	If I suspect a specific implant, I replace it with a shorter one.
	If I suspect a specific implant, I remove it.
	I initiate corticosteroid treatment without surgical intervention.
	I initiate vitamin B_12_ treatment without surgical intervention.
	I do not perform any medical/surgical treatment, but I monitor the patient.

**Table 2:** Demographic data of participants.

Parameter		Number of participants
Age	22-30	525
	31-40	647
	41-50	206
	51-65	71
	65+	28
Experience (years)	< 1	128
	1-5	269
	6-10	333
	11-15	271
	>15	476
Specialty	No specialty	354
	Endodontics	135
	Oral and maxillofacial radiology	72
	Oral and maxillofacial surgery	268
	Pediatric dentistry	112
	Periodontology	253
	Prosthodontic dentistry	159
	Restorative dentistry	124
Workplace	State oral and dental health center	62
	University hospital	529
	Private practice	886

**Table 3:** Choices of action in implant surgery for high-risk patients regarding nerve damage.

Choice of action	Number of participants (%)
Use of short implants	1224 (82.9%)
Evaluate tomographic images	1205 (81.6%)
Use of drill-stoppers intraoperatively	834 (56.5%)
Use of perioperative corticosteroids	211 (14.3%)
Use of navigation systems or guided surgical techniques	23 (15.6%)
Refer patient to an oral and maxillofacial surgeon	40 (2.7%)
Apply nerve repositioning or trans-positioning	61 (4.1%)

**Table 4:** Action map followed by the participants according to the survey.

Action map			n (%)
Prevention Stage	Third molar surgery	Not to perform coronectomy	81%
	Implant surgery	Use of short implants	82.9%
		Evaluate tomographic images	81.6%
Management Stage	After tooth extraction	Prescribe vitamin B_12_	74%
		Prescribe NSAIDs	71%
	After implant surgery	Prescribe vitamin B_12_	22%
		Prescribe corticosteroids	21%
